# Burnout and work satisfaction are differentially associated in gastroenterologists in Germany

**DOI:** 10.12688/f1000research.110296.3

**Published:** 2022-06-22

**Authors:** Charles Christian Adarkwah, Joachim Labenz, Oliver Hirsch

**Affiliations:** 1 Department of General Practice and Family Medicine, Philipps-University, Marburg, 35043, Germany; 2 CAPHRI School for Public Health and Primary Care, Department of Health Services Research, Maastricht University, Maastricht, The Netherlands; 3 Medizinische Klinik, Diakonie Klinikum, Siegen, 57074, Germany; 4 Bundesverband Gastroenterologie Deutschland (BVGD) e. V., Berlin, 10707, Germany; 5 Department of Psychology, FOM University of Applied Sciences, Siegen, 57078, Germany

**Keywords:** work satisfaction, risk of professional burnout, endoscopy, correlation of data, factor analysis

## Abstract

Background:

Burnout in the field of gastroenterology is an under-researched phenomenon. So far, only a few studies have dealt with this topic. There are large geographical variations in burnout rates with 16–20% of gastroenterologists in Mexico and Germany being at risk or having burnout, 30–40% in the United Kingdom, and 50–55% in South Korea, Canada, and the USA. The investigation of differential associations of burnout with important factors in gastroenterologists leading to tailored therapy recommendations is lacking. Therefore, we investigated the associations between work satisfaction and burnout in this specialization.

Methods:

We distributed an electronic survey to gastroenterologists organized mainly in the Federal Organization of Gastroenterology in Germany (the BVGD - Bundesverband Gastroenterologie Deutschland). The Maslach Burnout Inventory (MBI) and the Work Satisfaction Questionnaire (WSQ) were examined regarding their postulated internal structure in our sample of gastroenterologists. Canonical correlations were performed to examine the association between work satisfaction and burnout in endoscopy physicians.

Results:

An acceptable model fit was shown for both the MBI and the Work Satisfaction Questionnaire. The canonical correlation analysis resulted in two statistically significant canonical functions with correlations of .62 (p<.001) and .27 (p<.001). The full model across all functions was significant (χ ^2 ^(18) = 386.26, p<.001). Burden, personal rewards, and global item regarding the job situation were good predictors for less exhaustion, while patient care and professional relations were good predictors for personal accomplishment. This supports the recognition of burnout as being a multidimensional construct which has to be thoroughly diagnosed.

Conclusions:

Specific interventions should be designed to improve symptoms of burnout in endoscopy physicians according to their individual complaints as burnout is a multidimensional construct. Differential interventions should be offered on the basis of our study results in order to alleviate the issue of work satisfaction and burnout in endoscopy physicians.

## Introduction

Gastroenterology is a part of internal medicine. Unlike many other parts of internal medicine, it combines both cognitive aspects of medicine and medical interventions, i.e. endoscopy. The vast majority of gastroenterologists perform procedures, e.g. colonoscopy, endoscopic retrograde cholangiopancreatography or liver biopsy. These procedures have a potential risk of complications. They imply the risks of adverse events, missed diagnoses or misdiagnoses. In essence, this field can be compared to surgery regarding procedural stresses, in contrast to non-procedure based internal medicine subspecialties. ^1^ Important insights regarding burnout in gastroenterology can be concluded from examining both interventional and non-interventional gastroenterology. ^1^ Poor job satisfaction is an increasing issue in physicians in Germany. It is well known that low work satisfaction as well as high levels of stress can lead to symptoms of burnout ^2^ that have been noticed in up to 45% of physicians. ^3^ High levels of burnout can have negative effects on physicians’ health status, job performance, and patient satisfaction. ^4^ Burnout in the field of gastroenterology is an under-researched phenomenon. So far, only a few studies have dealt with this topic.

Endoscopy personnel should receive more recognition, their work environment should be improved, and they should have better job promotion. ^5^ In their systematic review regarding the prevalence of burnout in gastroenterologists, Ong *et al*. found work volume, heterogeneous age groups, and female gender to be the most frequently reported risk factors for higher levels of stress and burnout. ^6^ Gleeson *et al*. found significant stress levels in 20% of UK gastroenterologists which was associated with impaired health and suboptimal patient care. ^7^ Excessive work was found to be the main cause of high stress, also working conditions beyond control and conflict. Women were more susceptible to stress. Happiness with work was an important protective factor, relief from some duties and mentoring were perceived as possible solutions. Overall burnout prevalence in UK gastroenterology trainees measured by the MBI was 35.3% with more than half of the participants experiencing emotional exhaustion. ^8^ High workload, inadequate staffing levels, and interpersonal problems with colleagues were the most prominent stressors. In a sample of 411 Mexican gastroenterologists, an overall burnout prevalence of 26.3% was observed. Lack of support in case of complications, frequent reprimands from superiors, nonmedical duties during work, harassment/workplace violence were important factors associated with burnout. ^4^ Half of gastroenterologists in a survey in the USA reported burnout measured by the Maslach Burnout Inventory (MBI). Important factors associated with burnout were female gender, younger age, childless or younger children. ^9^ An international survey amongst 770 endoscopy trainees from 63 countries revealed a burnout rate of 18.8% which was measured with a single-item burnout scale. There was a positive correlation between burnout and anxiety severity. ^10^ Gastroenterologists, Surgeons, Radiologists, and Oncologists working as hospital consultants took part in a survey regarding burnout and psychiatric morbidity. ^11^ Radiologists had lower personal accomplishment scores, on all other scales of the MBI there were no significant differences. Feeling overloaded, and its effect on home life, feeling poorly managed and resourced, and dealing with patients’ suffering were associated with burnout. Problematic relationships with patients, relatives, and staff, low satisfaction with professional status/esteem, and low intellectual stimulation, younger than 55 years of age, and being single were all associated with burnout. Furthermore, those consultants who considered themselves to be not adequately trained in communication and management skills were also associated with burnout. Autonomy and self-management skills could be approaches for improvement. The development of standards assessing the performance of physicians judged without their participation undermines professional morale and may further increase the risk of burnout. ^11^This is further emphasized by Barnes *et al*. who cite results which show that young gastroenterologists are at higher risk of developing burnout. ^12^ Stress and burnout levels were moderately elevated in gastroenterologists, and it was demonstrated that younger interventional gastroenterologists with fewer years of experience showed higher stress and burnout levels. This might be caused by the complexity of procedures and by the fear of misinterpreting important findings. ^1^ In a previous publication of our representative sample of German gastroenterologists we were able to demonstrate relevant differences regarding burnout risk and job satisfaction. Younger physicians had significantly higher depersonalization and exhaustion scores with almost medium and small effect sizes. Those having a higher position in the clinic had higher accomplishment scores in the Maslach Burnout Inventory (MBI).

Physicians with more years of work were more satisfied in terms of patient care. Nevertheless, 17 % had high exhaustion scores, about 30% of our sample showed high depersonalization scores, and approximately half revealed low personal accomplishment scores. This altogether results in a higher general burden among German gastroenterologists. ^13^

To the best of our knowledge, no studies for the German setting are available to date examining differential associations between burnout and work satisfaction in physicians working in endoscopy units. With this study, we aim to investigate these differential associations and to extract predictors for burnout in the area of work satisfaction which can inform the design of future interventions.

## Methods

### Design and sample recruitment

The design and sample recruitment are already described in a previous publication. ^13^ The description is presented again here. We performed an online survey using the platform Limesurvey Version 3 for research institutions, universities and other educational institutions. Written informed consent was obtained from all individual participants included in the study. Physicians were queried about their baseline demographic variables, work satisfaction and their risk of burnout.

This survey was performed among gastroenterologists in Germany between January and April 2019. All members of the Federal Association of Gastroenterology in Germany (BVGD – Bundesverband Gastroenterologie Deutschland e.V.) were invited to take part in the study. The vast majority of physicians working in the field of gastroenterology, i.e. from residents to department heads in clinics, as well as physicians in private practice, hold a membership in this organization (n=3142). Participation in the survey was voluntary, anonymized and not incentivized.

The survey was comprised of an invitation with a detailed study description, an informed consent form and the study questionnaire. The German versions of the Work Satisfaction Questionnaire (WSQ-D) and the Maslach Burnout Inventory (MBI-D) were used in the study. Members of the BVGD received an email invitation to participate and a link to the study was also published on the BVGD website. After eight weeks a reminder to participate in the study was sent to all members. The study was conducted according to the guidelines of the Declaration of Helsinki and approved by the data protection commissioner of the University of Siegen and the Ethics Committee of the University of Essen Medical School (16-7125-BO). Informed written consent was given by all participants. ^13^

### Assessment of work satisfaction and risk of burnout

The Work Satisfaction Questionnaire - which was also described in a previous publication ^14^ - is comprised of 17 items to be scored on a seven-point scale from “1 – very dissatisfied” to “7 – very satisfied”. ^15^^,^^16^ The questionnaire was constructed based on the main components of work satisfaction which were identified by qualitative research conducted by the Society of General Internal Medicine Career Satisfaction Study Group. ^17^ The items of the questionnaire address satisfaction with overall quality of care, current income, type of payment mechanism, respect and prestige, enjoyment of work, continuing medical education opportunities, intellectual stimulation at work, autonomy to refer patients to a specialist, autonomy in treating patients, administrative burden, workload and work stress, time for family, friends or leisure, relationships with patients, peers, nurses and other non-medical staff, and job satisfaction in general. It has a five-factor structure, comprised of patient care (four items, Cronbach’s-α = .76), burden (four items, α = .79), income-prestige (three items, α = .83), personal rewards (three items, α = .71), and professional relations (two items, α = .66). Furthermore, a global item asks for the respondent’s satisfaction with their current job situation. This item correlates with the subscale scores from .39 – .71. ^15^ The instrument was shown to be sensitive to structural changes in healthcare systems. ^18^ The five-factor structure was supported in our study of GPs in a rural area of Germany by a confirmatory analysis. ^14^

We used the German version of the Maslach Burnout Inventory (MBI) to assess occupational burnout which was also described in another previous publication. ^19^ The MBI is designed in order to measure an enduring state of experiencing burnout, an assumption that is borne out by the stability of its scores over time. ^20^ The MBI is comprised of 22 items, each scored on a seven-point scale from “0 – never” to “7 – every day”. It consists of three subscales, namely “emotional exhaustion” (nine items) which measures exhaustion at work, “depersonalization” (five items), which measures emotional distance to others and loss of empathy, and “personal accomplishment” (eight items), which measures competence and a positive attitude towards work. The three-factor structure was confirmed: the Cronbach’s-α of the emotional exhaustion scale was .85, of the personal accomplishment subscale .71, and of the depersonalization subscale just .48. ^21^ Other studies found higher internal consistencies for this subscale with Cronbach’s-alphas of .69 and .86, respectively. ^22^^,^^23^ Convergent and discriminant validity of the MBI could be demonstrated. The three-factor structure was also supported by our study of German GPs. ^14^

### Statistical analyses

The following statistical measures were also applied in a previous publication. ^19^ We used chi-square tests with effect size Cramér’s V for comparing categorical variables and the Welch test with effect size Cohen’s d for comparing independent groups. ^24^^,^^25^

We conducted confirmatory factor analysis with the R package lavaan 0.6-7 ^26^ to examine the hypothesized factorial structures of the MBI and the Work Satisfaction Questionnaire in our sample of gastroenterologists. We used the robust Unweighted Least Squares Estimator (ULSMV), as this estimation method makes no distributional assumptions. ^26^^,^^27^ Different model-fit statistics were calculated. The χ ^2^/df ratio is a badness-of-fit-index as smaller values indicate a better fit. ^28^ Values between 2 and 5 signal an acceptable model fit. ^29^^,^^30^ The Root Mean Square Error of Approximation (RMSEA) is a population-based index that relies on the noncentral χ ^2^ distribution. It can be regarded as an “error of approximation” index because it assesses the extent to which a model fits reasonably well in the population. ^31^ Values ≤ .08 are considered to indicate an adequate model fit. ^32^ The standardized root mean square residual (SRMR) was calculated to measure the mean absolute value of covariance residuals. ^33^ Values below .10 indicate a good model fit. ^34^ The Comparative Fit Index (CFI) and the Tucker Lewis Index (TLI) were not considered as it was observed that they were sensitive to models with more variables than ours. ^35^ The resulting items and scales were examined by parameters based on classical test theory like Cronbach’s-α, discriminatory power, and average intercorrelations. Omega coefficients for the applied scales were also computed using R packages psych 2.0.7 (RRID:SCR_021744) and GPArotation 2014.11-1 as they have known advantages over Cronbach’s-α. ^36^

We used canonical correlation analysis to examine the association between work satisfaction and burnout in gastroenterologists. ^37^ According to previous findings we labelled the five scales and the overall item of the Work Satisfaction Questionnaire as independent variables and the three scales of the Maslach Burnout Inventory as dependent variables. The subject to variable ratio was 76 to 1 and therefore much higher than the recommended 10 to 1 ratio. ^38^ R packages yacca 1.4 (RRID:SCR_021746) and yhat 2.0-3 were used for calculations. All calculations were performed using R version 4.0.2 (R Project for Statistical Computing, RRID:SCR_001905). The underlying data can be found at Figshare. ^39^

## Results

### Study sample

Referring to the whole population of BVGD members, 22% of them took part in our study, resulting in a sample of 683 gastroenterologists. Of those, 508 were male (74.4%). The sample characteristics were compared to membership characteristics of the Federal Association of Gastroenterology in Germany as these members are considered to be a representative sample for the population of gastroenterologists in Germany. The single-sample chi-square test shows a significant result (χ ^2^ (1) = 18.67, p = .0000155), but the effect size Cramér’s V signals a small effect with .06. In conclusion, our sample should still be representative regarding gender. The mean age of the participants was 48.3 years (SD 9.1), with a median of 48 years, a minimum age of 27 and a maximum age of 75 years. Our sample was significantly older than the population (mean 44.4, SD 9.4; Welch-Test (df = 1006), t = -10.09, p < .001). Cohen’s d signals almost a medium effect with 0.42. This might be explained by an age effect. Members young in age and residents who are early in their training in this field have not yet gained endoscopy experience and do not fulfil inclusion criteria, because we focus explicitly on physicians working in endoscopy units. ^13^

Participants were working in the field of endoscopy on average for 16.5 years (SD 9.5). Most of the physicians were specialists in internal medicine and gastroenterology (n = 547, 80.1%). Exactly 500 (73.2%) were working in a hospital while 183 (26.8%) were working in practice. In the population, 94.3% are working in a hospital while 5.7% are working in practice. In comparison with the population, significantly more physicians in our sample work in practice (χ ^2^ (1) = 294.58, p < .0001). The effect size of Cramér’s V signals a medium effect with .27. ^13^

### Methodological evaluation of the Maslach Burnout Inventory (MBI)

We tested the hypothesized three-factor structure in our sample of gastroenterologists. The confirmatory factor analysis with the robust ULSMV estimation method showed an acceptable model fit: χ ^2^/df = 4.45, RMSEA = .071, SRMR = .072.

One item has a factor loading under the recommended cut-off value of .30. ^31^ It is item four (“I can easily understand how my colleagues feel about things”) of the factor personal accomplishment with a loading of .07. All other items have loadings between .35 and .88.

Intercorrelations of factors are satisfactory with emotional exhaustion correlating with depersonalization/loss of empathy by r = .73 and with personal accomplishment by r = -.48 while depersonalization/loss of empathy correlates with personal accomplishment by r = -.41.

The Cronbach’s-α coefficient of the emotional exhaustion subscale was .88, the omega coefficient was .88, and the average inter-item correlation was .45. The discriminatory power of the items ranged from .46 – .79. The Cronbach’s-α coefficient of the depersonalization/loss of empathy subscale was .75, the omega coefficient was .77, and the average inter-item correlation was .38. Discriminatory power of the items ranged from .27 – .67. The Cronbach’s-α coefficient of the personal accomplishment subscale was .77, the omega coefficient was .78, and the average inter-item correlation was .30. Discriminatory power of the items ranged from .23 (item four) – .59. All values can be classified as satisfactory to high except for the low discriminatory power of item four.

The mean of the scale emotional exhaustion was 16.5 (SD 10.1) with a median of 14, a minimum of 0, and a maximum of 48. In the main its distribution deviated significantly from a normal distribution: Shapiro-Wilk test, p < .0001; Skewness, p = .0001 (right-skewed); Kurtosis, p = .48. According to normative values published in Soler *et al*., ^40^ 311 physicians (45.5%) had a low level of emotional exhaustion, 259 (37.9%) an average level, and 113 (16.6%) reported a high level of emotional exhaustion.

The mean of the scale depersonalization/loss of empathy was 6.8 (SD 5.7); Huber’s M estimator was 6.2 with a median of 5, a minimum of 0, and a maximum of 28. Its distribution mainly deviated significantly from a normal distribution: Shapiro-Wilk test, p < .0001; Skewness, p < .0001 (right-skewed); Kurtosis, p = .002. According to normative values published by Soler *et al*., ^40^ 349 physicians (51.1%) had a low level of depersonalization/loss of empathy, 145 (21.2%) an average level, and 189 (27.7%) reported a high level of depersonalization/loss of empathy.

The mean of the scale for personal accomplishment was 32.5 (SD 8.3), with a median of 33, a minimum of 0, and a maximum of 48. Its distribution mainly deviated from a normal distribution: Shapiro-Wilk test, p < .0001; Skewness, p < .0001 (left-skewed); Kurtosis, p = .07. According to normative values published by Soler *et al*., ^40^ 154 physicians (22.5%) had a high sense of personal accomplishment, 161 (23.6%) an average level, and 361 (53.9%) reported a low level of personal accomplishment. This result might underestimate the sense of personal accomplishment of gastroenterologists as 100 (14.6%) physicians were just below the cut-off in the range of 31–33 points.

### Methodological evaluation of the Work Satisfaction Questionnaire

We tested the hypothesized five-factor structure in our GP sample. The confirmatory factor analysis with the robust ULSMV estimation method showed an acceptable model fit: χ ^2^/df = 4.97, RMSEA = .076, SRMR = .055.

All items had factor loadings over the recommended cut-off value of .30. ^31^ The range was between .38 and .89.

Intercorrelations of factors were heterogenous. The majority were in the moderate range while the intercorrelations between factors personal rewards, patient care, and professional relations were high (.83 – .94) ( Table 1).

**Table 1.** Factor intercorrelations of factors of the Work Satisfaction Questionnaire in the confirmatory factor analysis.

	Burden	Income-prestige	Personal rewards	Professional relations
Patient care	.28	.66	.83	.90
Burden		.41	.43	.27
Income-prestige			.74	.70
Personal rewards				.94

The Cronbach’s-α coefficient of the patient care subscale was .86; the omega coefficient was .86, and the average inter-item correlation was .60. Discriminatory power of the items ranged from .65 – .76. The Cronbach’s-α coefficient of the burden subscale was .86; the omega coefficient was .87, and the average inter-item correlation was .61. Discriminatory power of the items ranged from .57 – .79. The Cronbach’s-α coefficient of the income-prestige subscale was .69; the omega coefficient was .73, and the average inter-item correlation was .43. Discriminatory power of the items ranged from .44 – .63. The Cronbach’s-α coefficient of the personal rewards subscale was .81; the omega coefficient was .81, and the average inter-item correlation was .59. Discriminatory power of the items ranged from .62 – .69. The Cronbach’s-α coefficient of the professional relations subscale was .75, and the omega coefficient was .75; both items correlated by r = .60.

The mean of the scale for patient care was 21.8 (SD 4.7) with a median of 23, a minimum of 4, and a maximum of 28. Its distribution deviated from a normal distribution: Shapiro-Wilk test, p < .0001; Skewness, p < .0001 (left-skewed); Kurtosis, p < .0001 (leptokurtic). There were no significant differences in comparison with our GP sample (Welch-Test, t(120) = 0.18, p = .86, Cohen’s d = 0.02). ^14^

The mean of the scale for burden was 15.4 (SD 2.7) with a median of 16, a minimum of 9, and a maximum of 21. In the main its distribution corresponded to a normal distribution: Shapiro-Wilk test, p = .08; Skewness, p = .006 (right-skewed); Kurtosis, p = .0005 (platykurtic). Our GP sample reported a significantly higher satisfaction in this area with a medium effect size (Welch-Test, t(115) = -5.54, p < .001, Cohen’s d = 0.56). ^14^

The mean of the scale for income-prestige was 13.4 (SD 3.7) with a median of 14, a minimum of 3, and a maximum of 21. In the main its distribution deviated from a normal distribution: Shapiro-Wilk test, p < .0001; Skewness, p = .0001 (left-skewed); Kurtosis, p = .08. Our GP sample reported significantly higher satisfaction in this area with a medium effect size (Welch-Test, t(126) = -6.10, p < .001, Cohen’s d = 0.56). ^14^

The mean of the scale for personal rewards was 15.3 (SD 4.0) with a median of 16, a minimum of 3, and a maximum of 21. Its distribution deviated from a normal distribution: Shapiro-Wilk test, p < .0001; Skewness, p < .0001 (left-skewed); Kurtosis, p < .0001 (leptokurtic). Our GP sample reported significantly higher satisfaction in this area with almost a medium effect size (Welch-Test, t(138) = -5.38, p < .001, Cohen’s d = 0.45). ^14^

The mean of the scale for professional relations was 10.7 (SD 2.5) with a median of 11, a minimum of 2, and a maximum of 14. Its distribution deviated from a normal distribution: Shapiro-Wilk test, p < .0001; Skewness, p < .0001 (left-skewed); Kurtosis, p < .0001 (leptokurtic). Our GP sample reported significantly higher satisfaction in this area with a small effect size (Welch-Test, t(122)= -2.56, p = .01, Cohen’s d = 0.24). ^14^

### Association between burnout and work satisfaction

The canonical correlation analysis resulted in three canonical functions with canonical correlations of .62 (p < .001), .27 (p < .001), and .10 (p = .12). The full model across all functions was significant (χ ^2^ (18) = 386.26, p < .001). ^41^ The first two functions are statistically significant and the first function accounts for a considerable amount of variance (38% versus 7.5%, respectively). It is debated if squared multiple correlations are representing the amount of shared variance between two variable sets. ^42^ Cramer and Nicewander ^43^ proposed the average squared multiple correlation as the measure of variance accounted for which in our case would result in a shared variance of 15.5% and is according to standards in the behavioural and life sciences still in the acceptable range. ^44^

**Figure 1.**
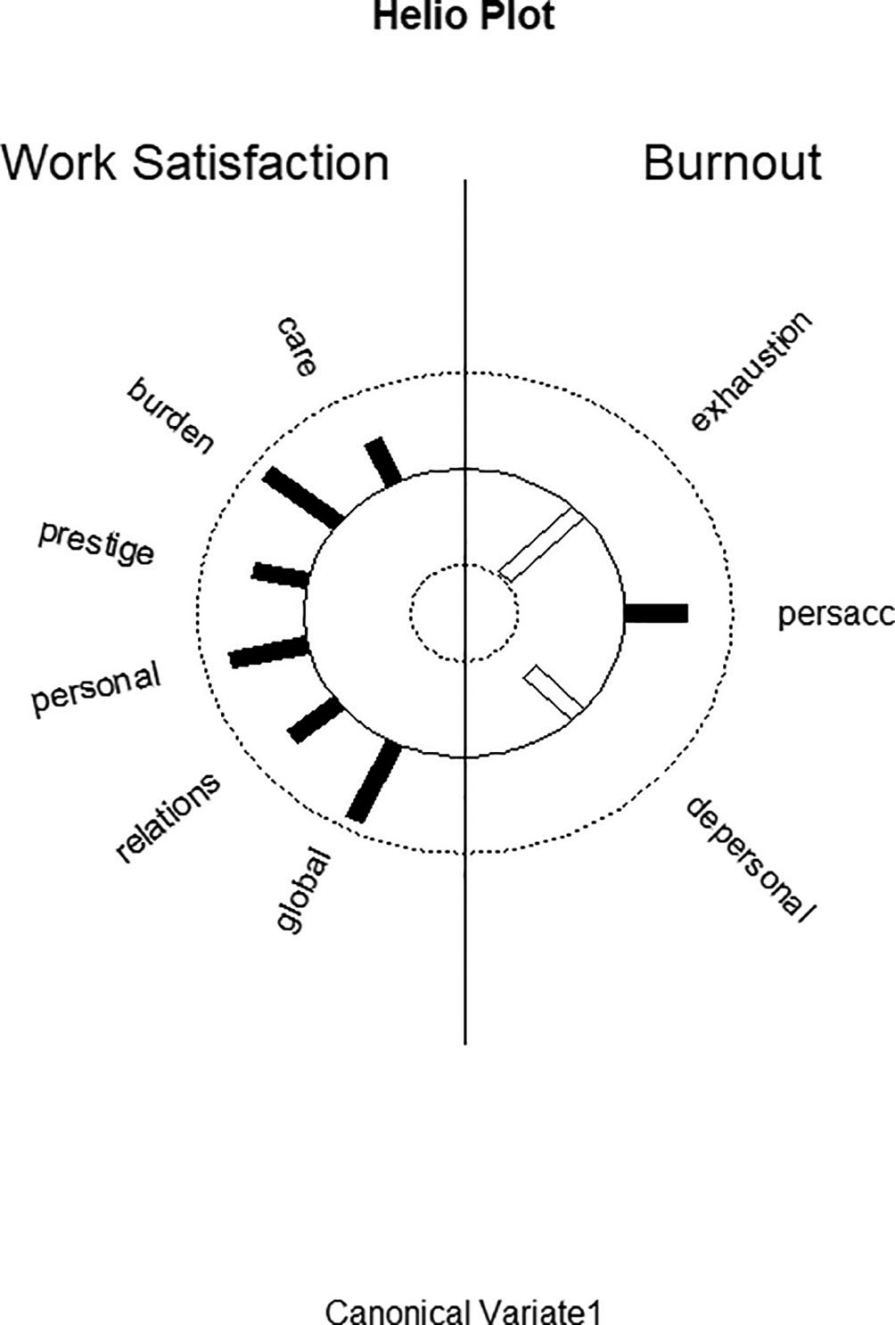
Graphic display of structure correlations (loadings) of the WSQ (Work Satisfaction Questionnaire) scales on the first predictor canonical variate and of the structure correlations (loadings) of the MBI (Maslach Burnout Inventory) scales on the first criterion canonical variate. Black bars correspond to positive correlations; white bars correspond to negative correlations.

**Function 1** The first criterion canonical variate is mainly characterized by exhaustion (r=-.96) which explains 92% of the variance of this variate ( Table 2). There is also a higher cross-loading of depersonalisation/lack of empathy which has its highest value in function 3, which did not reach significance. Burden, personal rewards, and the global item regarding the job situation seem to be particularly good predictors for less exhaustion, as exhaustion has a negative correlation with the first criterion canonical variate ( Table 2). This means that the more satisfied the gastroenterologists were with burden, personal rewards, and with their job situation in general, the less exhausted they felt. The low standardized function coefficients of income-prestige and professional relations and their relatively high correlations with the first canonical variate indicates that the variance of this variable is explained by the other variables. The predictor canonical variate is characterized by burden, personal rewards, and the global item regarding the job situation in general ( Table 3). Prestige displays a pattern of cross-loadings with similar correlations with functions 1 and 2 but a slightly higher value in function 1 ( Tables 3 and 4). Figure 1 displays the structure correlations (loadings) of the WSQ scales on the first predictor canonical variate and of the structure correlations (loadings) of the MBI scales on the first criterion canonical variate and visualizes the differential loading patterns and associations between job satisfaction and burnout variables in function 1.

**Table 2.** Standardized canonical coefficients and structure correlations of the first criterion canonical variate.

Criterion canonical variate	Standard canonical coefficients	Structure correlations
Emotional exhaustion	-0.86	-0.96
Personal accomplishment	0.31	0.57
Depersonalization	-0.01	-0.64

**Table 3.** Standardized canonical coefficients and structure correlations of the first predictor canonical variate.

Predictor canonical variate	Standard canonical coefficients	Structure correlations
Patient care	-0.19	0.48
Burden	0.52	0.85
Income-prestige	-0.01	0.52
Personal rewards	0.27	0.73
Professional relations	0.04	0.55
Global item	0.49	0.89

**Table 4.** Standardized canonical coefficients and structure correlations of the second predictor canonical variate.

Predictor canonical variate	Standard canonical coefficients	Structure correlations
Patient care	0.51	0.72
Burden	-0.45	-0.33
Income-prestige	0.31	0.45
Personal rewards	0.21	0.54
Professional relations	0.51	0.72
Global item	-0.53	0.26

**Figure 2.**
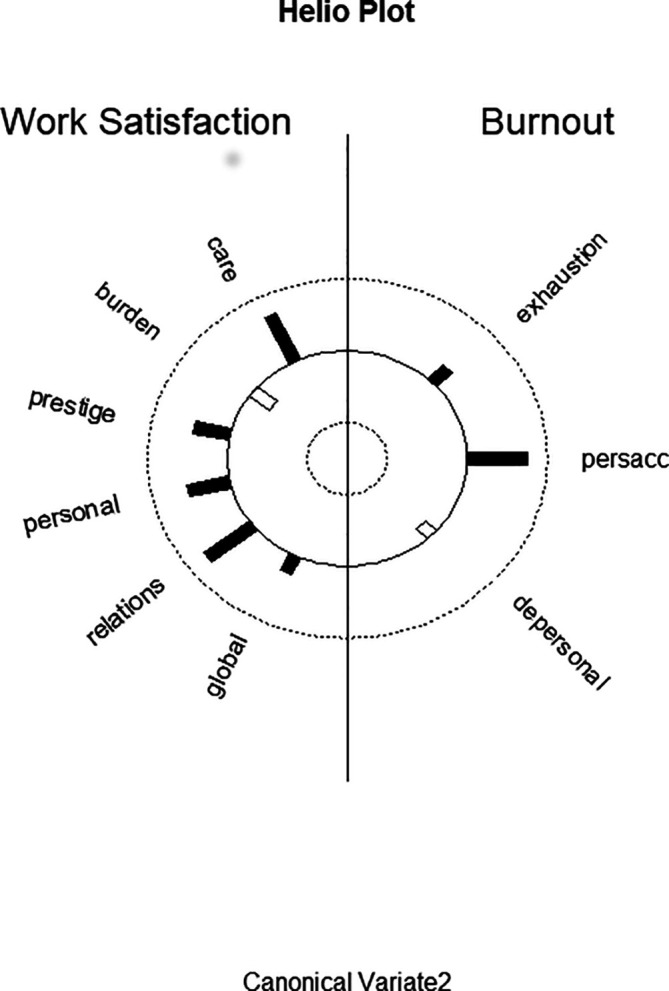
Graphic display of structure correlations (loadings) of the WSQ (Work Satisfaction Questionnaire) scales on the second predictor canonical variate and of the structure correlations (loadings) of the MBI (Maslach Burnout Inventory) scales on the second criterion canonical variate.

**Function 2** Patient care and professional relations seem to be good predictors for personal accomplishment. The predictor canonical variate is characterized by patient care and professional relations and the above-mentioned cross-loading of income/prestige ( Table 4). The second criterion canonical variate is characterized mainly by personal accomplishment (r = .76), which explains 57% of the variance of this variate ( Table 5). Figure 2 displays the structure correlations (loadings) of the WSQ scales on the second predictor canonical variate and of the structure correlations (loadings) of the MBI scales on the second criterion canonical variate. It visualizes the differential loading patterns and associations between job satisfaction and burnout variables in function 2. In relation to the other variables there is also a higher loading of personal relations, but this WSQ scale has a higher loading on the first predictor canonical variate.

**Table 5.** Standardized canonical coefficients and structure correlations of the second criterion canonical variate.

Criterion canonical variate	Standard canonical coefficients	Structure correlations
Emotional exhaustion	0.86	0.27
Personal accomplishment	0.88	0.76
Depersonalization	-0.50	-0.19

The third canonical correlation was low not significant, with .10 (p = .12). Therefore, the third function should not be interpreted.

## Discussion

We could demonstrate acceptable model fits in confirmatory factory analyses regarding the measurement of work satisfaction and burnout in gastroenterologists. These results are of importance, as the theoretical structure of questionnaires has to be confirmed in special subpopulations before respective subscores can be calculated. ^31^

We were able to confirm the three-factor structure of the Maslach Burnout Inventory in our sample. Item four (“I can easily understand how my colleagues feel about things”) of the personal accomplishment subscale had a factor loading < .30 and might be eliminated in this special subgroup. The internal consistency of the exhaustion subscale was high, while the internal consistencies of the depersonalization/loss of empathy and personal accomplishment subscales were satisfactory. According to norms published by Soler *et al*., ^40^ 16.6% of gastroenterologists could be classified as showing high emotional exhaustion, 27.7% high depersonalization/loss of empathy, and 53.9% were showing low personal accomplishment, although the last result might be misleading due to 15% being close to the cut-off value. Nevertheless, qualitative studies should evaluate the reasons for these numbers. Our results contradict ^1^^,^^45^ but also corroborate earlier studies. ^12^ In the systematic review of Ong *et al*. median values for emotional exhaustion, depersonalization, and low personal accomplishment were 25.7%, 25.6%, and 45.1%, respectively. They observed geographical variations in burnout rates with 16–20% of gastroenterologists in Mexico and Germany being at risk or having burnout, 30–40% in the United Kingdom, and 50–55% in South Korea, Canada, and the USA. The definition for burnout, its operationalisation, and cut-off values within the same instruments varied across studies; standardisations should be implemented at each level for better comparability of study results. ^46^ A tri-criteria approach of burnout based on a subscale algorithm within the MBI is proposed by Ong *et al*. ^6^ (1) Emotional Exhaustion ≥27 together with Depersonalization ≥13: (n=60, 8.8% of our sample) (2) Emotional Exhaustion ≥27 together with Personal Accomplishment ≤31: (n=74, 10.8% of our sample), and (3) Emotional Exhaustion >27 together with Depersonalization >10 and Personal Accomplishment <33: (n=50, 7.3% in our sample). However, it should be critically noted that it is unclear to what extent the cut-off values mentioned there are valid for the special population of gastroenterologists. Using these combined criteria, burnout rates are lower which are expected due to the additive conditions.

Among the most important measures for improving their situation were taking care of themselves and delegating administrative tasks. Higher burnout scores were associated with long working hours, especially in surgeons, and in physicians early in their career. ^47^

The five-factor structure of the Work Satisfaction Questionnaire was also confirmed. Internal consistencies were satisfactory and comparable with those of the original publications, ^15^^,^^18^ except for the subscale “income-prestige” which has a Cronbach’s-α coefficient of .69 and an omega coefficient of .73. The Spearman intercorrelations between the subscale scores were between .24 and .65 and are close to those listed in the original publications. In our confirmatory analyses, factor intercorrelations between the factors, personal rewards, patient care, and professional relations, were high, which shows a possible overlap between these subconstructs in our sample. Work satisfaction should be regarded as a multi-dimensional construct which contains different aspects with different internal structures in special subsamples. ^14^

We applied canonical correlation analysis to examine the association between work satisfaction and burnout in gastroenterologists. The first canonical function revealed that burden, personal rewards, and the global item regarding the job situation were good predictors for less exhaustion. The second canonical function showed that patient care and professional relations were good predictors for personal accomplishment. Our results corroborate several other findings. Feeling of support from colleagues had a protective effect. ^4^ Job satisfaction in health care had a relevant association to interprofessional teamwork. ^48^ In a previous study with primary care physicians, burden and the global item in the WSQ were good predictors of emotional exhaustion, while patient care, personal rewards and professional relations were good predictors of depersonalization/lack of empathy. ^14^ These different results in different physician groups suggest differential associations within physician subgroups which have consequences for interventions.

Our results have important implications for the clinical management of burnout in gastroenterologists. Burnout is a multidimensional construct, which should be examined in a differentiated way with all its facets. Depending on the severity of the manifestations on the different factors, differentiated interventions should be designed and individually planned. As our results reveal, a gastroenterologist scoring high on emotional exhaustion would need a different intervention, as there are different associations with work satisfaction than for an gastroenterologists scoring low on personal accomplishment. Therefore, the results of our study could be used to design specific interventions to improve circumscribed symptoms of burnout. To date, several global interventions have been developed to reduce job stress and the risk of burnout in gastroenterologists. A psychoeducational intervention reduced burnout and anxiety symptoms in physicians in comparison with a control group, but other health and habit-related outcomes were unaffected, as they were measured just seven days after the end of the intervention. ^49^ The effects of problem-focused coping become evident as one engages in strategies to change the stressful situation. This was associated with lower levels of burnout, distress, and higher levels of job-related self-efficacy. Problem-focused coping strategies were more likely to be used by female gastroenterologists. Higher burnout scores were associated with emotion-focused coping. ^50^ The new specific interventions to be developed must first undergo an evidence-based evaluation process before they can be applied in individual cases. Long-term studies must then additionally show whether any effects remain stable. Quality in endoscopy is a complex construct. Accordingly, evaluation of such a complex construct is difficult, as interactions between endoscopy personnel, patients, cultural, and societal perspectives must all be considered. One can imagine that work satisfaction and burnout symptoms in endoscopy personnel can have a decisive influence on these subtle processes and consequently both can impact quality in endoscopy. ^51^ Increased job stress and burnout might result in suboptimal care, a higher rate of medical errors, and earlier retirement. ^1^ Distress negatively affects cognitive functioning and clinical decision-making ^52^ and puts patients at risk. ^53^ By addressing these areas, a contribution is made to improving care in the field of gastroenterology.

Our study has limitations that should be considered. Our response rate was 22% and could therefore have been higher, to gain a more complete understanding of the associations between work satisfaction and burnout. Nevertheless, this response rate is almost identical ^4^^,^^7^ or is considerably higher than in other studies in this area. ^1^^,^^9^^,^^50^ The survey was sent to members of a professional society which may not fully represent the population of gastroenterologists. We collected cross-sectional data based on self-reports which always have to be interpreted with caution. We had to refer to reference numbers from other specialties, mainly primary care, as German norms in work satisfaction and burnout are lacking in gastroenterology, but as these were European data, we think that they are more comparable than existing American norm data. It might have been that those physicians who are less exhausted by their work took the time for the survey. As a consequence, more severe cases of burnout risk might have been missed in the analysis and the predictive value of the fitted models might be better in mild cases of burnout risk. In a previous publication we examined possible confounding factors. Age and employment status were issues with small to medium effects regarding differences in burnout risk and work satisfaction. Elderly physicians were more satisfied with their work and had on average a lower burnout risk. Physicians in higher positions were more satisfied with their work and had a lower burnout risk. This should be taken into account when interpreting the results of the current analyses. ^13^

## Conclusions

Specific interventions should be designed to improve symptoms of burnout in gastroenterologists according to their individual complaints as burnout is a multidimensional construct. For example, gastroenterologists scoring high in emotional exhaustion need a different intervention than gastroenterologists scoring low in personal accomplishment, as each group of respondents has different associations regarding work satisfaction. Consequently, differential interventions should be offered on the basis of our study results in order to alleviate the issue of work satisfaction and burnout in endoscopy physicians.

## Data availability

### Underlying data

Figshare: Underlying data for ‘Burnout and work satisfaction are differentially associated in gastroenterologists in Germany’. https://doi.org/10.6084/m9.figshare.12144738.v6. ^40^

### Reporting guidelines

Figshare: STROBE checklist for ‘Burnout and work satisfaction are differentially associated in gastroenterologists in Germany’. https://doi.org/10.6084/m9.figshare.12144738.v6. ^40^

Data are available under the terms of the Creative Commons Attribution 4.0 International license (CC-BY 4.0)

## Consent

Written informed consent for publication of the participants’ details was obtained from the participants.
