# Supplementary material for: Burnout and work satisfaction are differentially associated in gastroenterologists in Germany
Source: F1000Res. 2022 Jun 22;11:368. Originally published 2022 Mar 30. [Version 3] doi: 10.12688/f1000research.110296.3 (PMC9152463; doi:10.12688/f1000research.110296.3)
Supplement: Peer review history

# Peer review: Reviewer response for version 3

**DOI:** 10.5256/f1000research.135333.r141678

**Published:** 2022-06-24

**Authors:** Amritpal Pali Hungin

**Affiliations:** 1 General Practice, Faculty of Medical Sciences, Newcastle University, Newcastle, UK

Approved.

Is the work clearly and accurately presented and does it cite the current literature?

Yes

If applicable, is the statistical analysis and its interpretation appropriate?

I cannot comment. A qualified statistician is required.

Are all the source data underlying the results available to ensure full reproducibility?

Yes

Is the study design appropriate and is the work technically sound?

Yes

Are the conclusions drawn adequately supported by the results?

Yes

Are sufficient details of methods and analysis provided to allow replication by others?

Yes

Reviewer Expertise:

NA

I confirm that I have read this submission and believe that I have an appropriate level of expertise to confirm that it is of an acceptable scientific standard.

# Peer review: Reviewer response for version 3

**DOI:** 10.5256/f1000research.135333.r141679

**Published:** 2022-06-22

**Authors:** Ralph Brinks

**Affiliations:** 1 Faculty of Health/School of Medicine, Witten/Herdecke University, Witten, Germany

I thank the authors for the clarifications and do not have any further comments.

Is the work clearly and accurately presented and does it cite the current literature?

Yes

If applicable, is the statistical analysis and its interpretation appropriate?

Partly

Are all the source data underlying the results available to ensure full reproducibility?

Yes

Is the study design appropriate and is the work technically sound?

Partly

Are the conclusions drawn adequately supported by the results?

Partly

Are sufficient details of methods and analysis provided to allow replication by others?

Partly

Reviewer Expertise:

Statistics, epidemiology, chronic diseases

I confirm that I have read this submission and believe that I have an appropriate level of expertise to confirm that it is of an acceptable scientific standard.

# Peer review: Reviewer response for version 2

**DOI:** 10.5256/f1000research.134623.r138625

**Published:** 2022-06-07

**Authors:** Ralph Brinks

**Affiliations:** 1 Faculty of Health/School of Medicine, Witten/Herdecke University, Witten, Germany

The manuscript ' *Burnout and work satisfaction are differentially associated in gastroenterologists in Germany*' by Adarkwah *et al.* reports on a voluntary online survey among members of the national association of gastroenterologists. Two major research questions are approached: 1) validation of two established questionnaires, Maslach Burnout Inventory (MBI) and the Work Satisfaction Questionnaire (WSQ), and 2) apply correlation analyses between MBI and WSQ.

 The authors address an important problem in patient care. Burnout and exhaustion are downsides in people caring for others, which can lead to enormous personal and occupational problems such as, for example, poor mental health, frequent sick leaves and increased error rates. In extreme cases, permanent sick leaves can lead to shutdown of health services.

 My biggest concern is the generalizability of the studies' findings. The authors use self-reports in a voluntary online survey. All members of the relevant association of gastroenterologist in Germany have been invited to take part. It has not been described how this invitation was accomplished (eg on the association's periodical, electronic newsletter, etc.) and whether members have been reminded in case they did not take part. It has not been described if any measures have been implemented to avoid double entries. About a fourth of the invited members took part in the online survey. Although the authors write that "the sample characteristics were compared to membership characteristics of the Federal Association of Gastroenterology in Germany as these members are considered to be a representative sample for the population of gastroenterologists" the reader does not learn which characteristics have been compared and what the results were in detail. Possibly, these findings can be added to the manuscript. Thus, even in case the members of the association are a representative sample of all gastroenterologists in Germany, the reader does not get to know details why the members who responded to the online survey are representative for the association's members. With a view to the relevant outcome of this study, it seems conceivable that only those physicians who are less exhausted by their work took the time for the survey. As a consequence, more severe cases of burnout might be missed in the analysis and the predictive value of the fitted models might be better in mild cases of exhaustion.

 The second biggest concern refers to confounding. The findings in the manuscript come from an observational study and by such are prone to possible confounding. As a reader of the study findings, I am interested in what has been done to reduce the effect of possible confounding. Unfortunately, this has not been described in the manuscript. The authors are aware about the limitations of voluntary self-report and state "We collected cross-sectional data based on self-reports which always have to be interpreted with caution", but it would be helpful for the readers if the authors could comment in which way and regarding which aspects the readers should be cautious.

 The authors shared the data in a public repository, which is excellent. A description of the variables would be helpful. The relevant reporting guideline (STROBE for cross-sectional studies) has been used. The uploaded data set occasionally contains missing values. In the main text, it has not been described how these have been treated during analysis. As the statistics software usually deletes subjects with missing values, I guess this is the method that has implicitly been used.

Is the work clearly and accurately presented and does it cite the current literature?

Yes

If applicable, is the statistical analysis and its interpretation appropriate?

Partly

Are all the source data underlying the results available to ensure full reproducibility?

Yes

Is the study design appropriate and is the work technically sound?

Partly

Are the conclusions drawn adequately supported by the results?

Partly

Are sufficient details of methods and analysis provided to allow replication by others?

Partly

Reviewer Expertise:

Statistics, epidemiology, chronic diseases

I confirm that I have read this submission and believe that I have an appropriate level of expertise to confirm that it is of an acceptable scientific standard, however I have significant reservations, as outlined above.

# Response

**Published:** 2022-06-20

**Authors:** Oliver Hirsch

**Affiliations:** FOM University of Applied Sciences, Germany

Comment: My biggest concern is the generalizability of the studies' findings. The authors use self-reports in a voluntary online survey. All members of the relevant association of gastroenterologist in Germany have been invited to take part. It has not been described how this invitation was accomplished (eg on the association's periodical, electronic newsletter, etc.) and whether members have been reminded in case they did not take part. It has not been described if any measures have been implemented to avoid double entries. About a fourth of the invited members took part in the online survey. Although the authors write that "the sample characteristics were compared to membership characteristics of the Federal Association of Gastroenterology in Germany as these members are considered to be a representative sample for the population of gastroenterologists" the reader does not learn which characteristics have been compared and what the results were in detail. Possibly, these findings can be added to the manuscript. Thus, even in case the members of the association are a representative sample of all gastroenterologists in Germany, the reader does not get to know details why the members who responded to the online survey are representative for the association's members. With a view to the relevant outcome of this study, it seems conceivable that only those physicians who are less exhausted by their work took the time for the survey. As a consequence, more severe cases of burnout might be missed in the analysis and the predictive value of the fitted models might be better in mild cases of exhaustion.

**Response: We thank the reviewer for the overall positive judgement of our piece of research and valuable comments on our manuscript. Here are the answers to the questions / issues raised:**

**We described the sampling process in Adarkwah et al. (2020), citation 13. Members of the BVGD received an email invitation to participate, and the study link was also published on the BVGD website. A reminder to participate in the study was sent after 8 weeks to all members. The demographic characteristics of the BVGD members were taken as comparison. The characteristics on which comparisons were performed can be found in the Results section under heading “study sample”:**

**“The single-sample chi-square test shows a significant result (χ 2 (1) = 18.67, p = .0000155), but the effect size Cramér’s V signals a small effect with .06. In conclusion, our sample should still be representative regarding gender. The mean age of the participants was 48.3 years (SD 9.1), with a median of 48 years, a minimum age of 27 and a maximum age of 75 years. Our sample was significantly older than the population (mean 44.4, SD 9.4; Welch-Test (df = 1006), t = -10.09, p < .001). Cohen’s d signals almost a medium effect with 0.42. This might be explained by an age effect. Members young in age and residents who are early in their training in this field have not yet gained endoscopy experience and do not fulfil inclusion criteria, because we focus explicitly on physicians working in endoscopy units.(::::)** **In comparison with the population, significantly more physicians in our sample work in practice (χ 2 (1) = 294.58, p < .0001). The effect size of Cramér’s V signals a medium effect with .27.”**

**We have added the mentioned possible selection mechanism regarding participation in our survey in the limitations.**

**We now refer more clearly to this earlier study where the study procedures are described in more detail.**

 The second biggest concern refers to confounding. The findings in the manuscript come from an observational study and by such are prone to possible confounding. As a reader of the study findings, I am interested in what has been done to reduce the effect of possible confounding. Unfortunately, this has not been described in the manuscript. The authors are aware about the limitations of voluntary self-report and state "We collected cross-sectional data based on self-reports which always have to be interpreted with caution", but it would be helpful for the readers if the authors could comment in which way and regarding which aspects the readers should be cautious.

**Response: In a previous publication we examined possible confounding factors (Adarkwah et al., 2020, citation 14). Age and employment status were issues with small to medium effects regarding differences in burnout risk and work satisfaction. Elderly physicians were more satisfied with their work and had on average a lower burnout risk. Physicians in higher positions were more satisfied with their work and had a lower burnout risk.** **We now refer more clearly to this earlier publication.**

 The authors shared the data in a public repository, which is excellent. A description of the variables would be helpful. The relevant reporting guideline (STROBE for cross-sectional studies) has been used. The uploaded data set occasionally contains missing values. In the main text, it has not been described how these have been treated during analysis. As the statistics software usually deletes subjects with missing values, I guess this is the method that has implicitly been used.

**Response: We thank the reviewer for this comment. A list of variables is uploaded in the repository. There were n=71 participants who discontinued the survey at a very early stage so that missing value replacement was not feasible and additionally a significant proportion of this group gave implausible data, e.g. discrepancy between age and years working in the field so that they had to be excluded. The remaining participants gave complete data on the questionnaires and the very few missing on demographic variables were deleted during analyses.**

# Peer review: Reviewer response for version 1

**DOI:** 10.5256/f1000research.121885.r129523

**Published:** 2022-05-30

**Authors:** Klaus Weckbecker

**Affiliations:** 1 Institute of General Practice and Primary Care, Faculty of Health, Witten/Herdecke University, Witten, Germany

Whether physicians are satisfied with their work and feel comfortable in their working environment is an important aspect. Dissatisfaction with the working conditions and even symptoms of burnout reduce the quality of the physician's performance. Thus, dissatisfaction with working conditions and the presence of a burnout syndrome endanger not only the health of the physicians concerned, but also the health of the patients entrusted to their care.

 The authors address this very interesting topic using the example of the specialist group of gastroenterologists in Germany. These doctors work in their own practices and are thus active both as business owners and as physicians. Another special feature of outpatient gastroenterologists in Germany is that they are active both interventively with endoscopies and in the long-term care of patients with gastrointestinal diseases.

 Thus, this work addresses a particularly interesting issue with high relevance for the health of practicing physicians as well as for the patients entrusted to their care.

 The authors provide an excellent summary of the current state of knowledge, taking into account the international literature.

 The chosen study design is appropriate to the research question. The chosen methodology of a cross-sectional survey by an online questionnaire is appropriate, the response rate and number of participants is good. Nevertheless, with this methodology there is always the risk that certain subgroups participated in the survey in excess. Here, for example, it would be conceivable that gastroenterologists in particular, who feel overwhelmed, frequently participate in the study. This is a weakness of the study that cannot be avoided due to the methodology, but which the authors also clearly state. Nevertheless, the results are highly relevant and should give rise to further research activities in this field. It would be particularly interesting to examine whether satisfaction with working conditions in other countries and / or under other economic conditions differs significantly from the data collected here.

 This interesting work can thus be the basis for further research projects, but also the trigger for discussions and reflections on our working conditions as physicians.

Is the work clearly and accurately presented and does it cite the current literature?

Yes

If applicable, is the statistical analysis and its interpretation appropriate?

Yes

Are all the source data underlying the results available to ensure full reproducibility?

Yes

Is the study design appropriate and is the work technically sound?

Yes

Are the conclusions drawn adequately supported by the results?

Yes

Are sufficient details of methods and analysis provided to allow replication by others?

Yes

Reviewer Expertise:

Family doctor, General practitioner

I confirm that I have read this submission and believe that I have an appropriate level of expertise to confirm that it is of an acceptable scientific standard.

# Response

**Published:** 2022-05-30

**Authors:** Oliver Hirsch

**Affiliations:** FOM University of Applied Sciences, Germany

Thank you very much for the kind consideration of our work. We really appreciate this. We have extended our evaluations of burnout to include the tri-criteria approach recommended by Ong et al (2021): (1) EE≥27 together with DP≥13, (2) EE≥27 together with PA≤31, and (3) EE>27 together with DP>10 and PA<33.

# Peer review: Reviewer response for version 1

**DOI:** 10.5256/f1000research.121885.r135853

**Published:** 2022-05-11

**Authors:** Amritpal Pali Hungin

**Affiliations:** 1 General Practice, Faculty of Medical Sciences, Newcastle University, Newcastle, UK

This an important piece of research in an increasingly important topic. Burnout and a lack of work satisfaction amongst clinicians is an international issue and is compromising patient care as well has causing health harm to clinicians themselves. In an area of ever increasing technological advances, increased patient and public expectations and the limited availability of health resource the situation is likely to worsen unless clearly recognised and accepted by the medical profession and the health authorities. Timely solutions need to be suggested.

 This manuscript captures the situation well, highlighting the relevant international literature and researching the situation in Germany amongst gastroenterologists using appropriate and accepted assessment tools. The authors suggest strategies for responding to the different, individual dimensions contributing to burnout as based on assessment tools and analysed data.

 The study design is appropriate for the research question, which is clearly set out: a study of the burnout and work satisfaction amongst gastroenterologists in Germany. The sampling frame is appropriate - the official professional group of gastroenterologists in Germany. Whilst the conventionally measured response rate might be considered low by some (22%) this is reasonable for such surveys and the overall numbers of respondents was high (683 gastroenterologists). The electronic survey method used is appropriate for a large, widely dispersed population. The responders were reasonably representative of the target population even though the authors recognise that the respondents’ age was somewhat skewed upwards.

 There is sufficient detail of methods and analysis to allow replication by others wishing to conduct similar studies and the statistical analysis appears comprehensive, even if somewhat complex to me personally. The interpretation of the data is appropriate although it is likely to be better understood by someone familiar with the questionnaires and their sub-domains. The source data and the results match up well.

 The conclusions are thoughtfully drawn. In addition to providing data on burnout and work satisfaction amongst this group in Germany, there are suggestions about the need to delineate and to tailor remedial actions based on specific subset findings from the survey. This is an important point in guiding interventions to help gastroenterologists who are struggling – the need to use problem focussed solutions to enhance coping strategies.

 This research represents an important step in better understanding the field of burnout and work satisfaction amongst gastroenterologists and I hope it will be more widely disseminated. It adds to the literature from other fields.

Is the work clearly and accurately presented and does it cite the current literature?

Yes

If applicable, is the statistical analysis and its interpretation appropriate?

I cannot comment. A qualified statistician is required.

Are all the source data underlying the results available to ensure full reproducibility?

Yes

Is the study design appropriate and is the work technically sound?

Yes

Are the conclusions drawn adequately supported by the results?

Yes

Are sufficient details of methods and analysis provided to allow replication by others?

Yes

Reviewer Expertise:

Primary/Secondary interface care, medical professionalism, gastroenterology, barriers to evidence based clinical care

I confirm that I have read this submission and believe that I have an appropriate level of expertise to confirm that it is of an acceptable scientific standard.

# Response

**Published:** 2022-05-30

**Authors:** Oliver Hirsch

**Affiliations:** FOM University of Applied Sciences, Germany

Thank you very much for the kind consideration of our work. We really appreciate this.
